# Supplementary material for: Mutations in dnaA and a cryptic interaction site increase drug resistance in Mycobacterium tuberculosis
Source: PLoS Pathog. 2020 Nov 30;16(11):e1009063. doi: 10.1371/journal.ppat.1009063 (PMC7738170; doi:10.1371/journal.ppat.1009063)
Supplement: S2 Fig — Alignment of DnaA protein sequence from Mycobacterium tuberculosis (MTB), Mycolicibacterium smegmatis (MSM), Escherichia coli (Ecoli) and Bacillus subtilis (Bsubt) with clustal omega as implemented on uniprot.org. Visualization was created in boxshade (https://embnet.vital-it.ch/software/BOX_form.html). Red indicates identity with MTB sequence. Blue indicated similarity with MTB sequence. Sites with similarity in 3 or 4 sequences are annotated in the consensus line with an upper case letter indicating 100% identity across all 4 sequences and lower case letter indicating at least one sequence without amino acid identity. (PDF) [file ppat.1009063.s002.pdf]

```
SP|P9WNW3|MTB       1 MTDDPGSGFTTVWNAVVSELNGDPKVDDGPSSDANLSAPLTPQQRAWLNLVQPLTIVEGF
SP|A0R7K1|MSM       1 MTADPDPPFVAVWNSVVAELNGDVNGDRQG---DPSLPVLTPQQRAWLKLVKPLVIAEGF
SP|P03004|Ecoli     1 -------MSLSLWQQCLARLQDE---------------LPATEFSMWIRPLQAE-LSDNT
SP|P05648|Bsubt     1 -----MENILDLWNQALAQIEKK---------------LSKPSFETWMKSTKAHSLQGDT
consensus           1            vWn  v  ln d                  p    Wl  v    i e

SP|P9WNW3|MTB      61 ALLSVPSSFVQNEIERHLRAPITDALSRRLGHQIQ-LGVRIA--PPATDEADDTTVPPSE
SP|A0R7K1|MSM      58 ALLSVPTPFVQNEIERHLREPIVTALSRKLGQRVE-LGVRIA--TPTDEPEDAPDSFADS
SP|P03004|Ecoli    38 LALYAPNRFVLDWVRDKYLNNINGLLTSFCGADAPQLRFEVGTKPVTQTPQAAVTS--NV
SP|P05648|Bsubt    41 LTITAPNEFARDWLESRYLHLIADTIYELTGEELS-IKFV----IPQNQDVE--------
consensus          61   ls P  Fv   ie      I   ls   G  i -l   ia-- p     d

SP|P9WNW3|MTB     118 NPATTSPDTTTDNDEIDDSAAARGDNQHSWPSYFTERPHNTDSATAGVTSLNRRYTFDTF
SP|A0R7K1|MSM     115 PAPASVPAGPADADEIDDDRDARVNAQESWPKYFSRPEPDTSSDDSNAVNLNRRYTFDTF
SP|P03004|Ecoli    96 AA-------PAQVAQTQPQ-RAAPSTRSGWDNVPA---P---AEPTYRSNVNVKHTFDNF
SP|P05648|Bsubt    88 ---------------------------DFMPKPQVKKAVKEDTSDFPQNMLNPKYTFDTF
consensus         121                      a       wp           s       lN ryTFDtF
                                  191               209                     233
SP|P9WNW3|MTB     178 VIGASNRFAHAAALAIAEAPARAYNPLFIWGESGLGKTHLLHAAGNYAQRLFPGMRVKYV
SP|A0R7K1|MSM     175 VIGASNRFAHAATLAIAEAPARAYNPLFIWGESGLGKTHLLHAAGNYAQRLFPGMRVKYV
SP|P03004|Ecoli   142 VEGKSNQLARAAARQVADNPGGAYNPLFLYGGTGLGKTHLLHAVGNGIMARKPNAKVVYM
SP|P05648|Bsubt   121 VIGSGNRFAHAASLAVAEAPAKAYNPLFIYGGVGLGKTHLMHAIGHYVIDHNPSAKVVYL
consensus         181 ViG sNrfAhAA laiAeaParAYNPLFiwG sGLGKTHLlHA Gny     P  rV Yv
                                                                 282            297
SP|P9WNW3|MTB     238 STEEFTNDFINSLRDDRKVAFKRSYRDVDVLLVDDIQFIEGKEGIQEEFFHTFNTLHNAN
SP|A0R7K1|MSM     235 STEEFTNDFINSLRDDRKASFKRSYRDIDILLVDDIQFIEGKEGIQEEFFHTFNTLHNSN
SP|P03004|Ecoli   202 HSERFVQDMVKALQNNAIEEFKRYYRSVDALLIDDIQFFANKERSQEEFFHTFNALLEGN
SP|P05648|Bsubt   181 SSEKFTNEFINSIRDNKAVDFRNRYRNVDVLLIDDIQFLAGKEQTQEEFFHTFNTLHEES
consensus         241 stE Ftndfinslrd r   Fkr YR vDvLLvDDIQFi gKE  QEEFFHTFNtLh   n

SP|P9WNW3|MTB     298 KQIVISSDRPPKQLATLEDRLRTRFEWGLITDVQPPELETRIAILRKKAQMERLAVPDDV
SP|A0R7K1|MSM     295 KQIVISSDRPPKQLATLEDRLRTRFEWGLITDVQPPELETRIAILRKKAQMDRLDVPDDV
SP|P03004|Ecoli   262 QQIILTSDRYPKEINGVEDRLKSRFGWGLTVAIEPPELETRVAILMKKADENDIRLPGEV
SP|P05648|Bsubt   241 KQIVISSDRPPKEIPTLEDRLRSRFEWGLITDITPPDLETRIAILRKKAKAEGLDIPNEV
consensus         301 kQIvisSDRpPK l tlEDRLrtRFeWGLitdv PPeLETRiAILrKKA  e l vP dV
                            365                                400
SP|P9WNW3|MTB     358 LELIASSIERNIRELEGALIRVTAFASLNKTPIDKALAEIVLRDLIADANTMQISAATIM
SP|A0R7K1|MSM     355 LELIASSIERNIRELEGALIRVTAFASLNKTRIDRSLAEVVLRDLIADATTMQISTAAIM
SP|P03004|Ecoli   322 AFFIAKRLRSNVRELEGALNRVIANANFTGRAITIDFVREALRDLLALQE-KLVTIDNIQ
SP|P05648|Bsubt   301 MLYIANQIDSNIRELEGALIRVVAYSSLINKDINADLAAEALKDIIPSSKPKVITIKEIQ
consensus         361 l  IA  ie NiRELEGALiRV Afasl    I   la   LrDlia      is   I
                      419                     443                     467
SP|P9WNW3|MTB     418 AATAEYFDTTVEELRGPGKTRALAQSRQIAMYLCRELTDLSLPKIGQAFG-RDHTTVMYA
SP|A0R7K1|MSM     415 AVTAEYFETTVEELRGPGKTRALAQSRQIAMYLCRELTDLSLPKIGQAFG-RDHTTVMYA
SP|P03004|Ecoli   381 KTVAEYYKIKVADLLSKRRSRSVARPRQMAMALAKELTNHSLPEIGDAFGGRDHTTVLHA
SP|P05648|Bsubt   361 RVVGQQFNIKLEDFKAKKRTKSVAFPRQIAMYLSREMTDSSLPKIGEEFGGRDHTTVIHA
consensus         421    aeyf   veelrg  ktr lA  RQiAMyL rElTd SLPkIG aFG RDHTTVm A
                            484     492
SP|P9WNW3|MTB     477 QRKILSEMAERREVFDHVKELTTRIRQRSKR
SP|A0R7K1|MSM     474 EKKIRGEMAERREVFDHVKELTTRIRQRAKR
SP|P03004|Ecoli   441 CRKIEQLREESHDIKEDFSNLIRTLSS----
SP|P05648|Bsubt   421 HEKISKLLADDEQLQQHVKEIKEQLK-----
consensus         481  rKI   mae  ev dhvkel   ir
```

Figure S2. Multispecies alignment of DnaA Alignment of DnaA protein sequence from Mycobacterium tuberculosis (MTB), Mycolicibacterium smegmatis (MSM), Escherichia coli (Ecoli) and Bacillus subtilis (Bsubt) with clustal omega as implemented on uniprot.org. Visualization was created in boxshade (https://embnet.vital-it.ch/software/BOX_form.html). Red indicates identity with MTB sequence. Blue indicated similarity with MTB sequence. Sites with similarity in 3 or 4 sequences are annotated in the consensus line with an upper case letter indicating 100% identity across all 4 sequences and lower case letter indicating at least one sequence without amino acid identity.
