# Supplementary material for: Mutations in dnaA and a cryptic interaction site increase drug resistance in Mycobacterium tuberculosis
Source: PLoS Pathog. 2020 Nov 30;16(11):e1009063. doi: 10.1371/journal.ppat.1009063 (PMC7738170; doi:10.1371/journal.ppat.1009063)
Supplement: S3 Fig — Alamar blue MIC measurements of indicated WT and dnaA mutant strains against INH (A), RIF (B), SM (C), and OFLX (D). Each point represents the mean and standard deviation among three independent clones for each genotype. The dashed line in each panel indicates the concentration used for competition assays in Fig 2. Differences among Alamar blue reduction (OD570) between WT and each mutant were tested using Dunnett’s multiple comparison test after 2-way ANOVA. * p < 0.05, ** p < 0.01, *** p < 0.001, **** p < 0.0001. (PDF) [file ppat.1009063.s003.pdf]

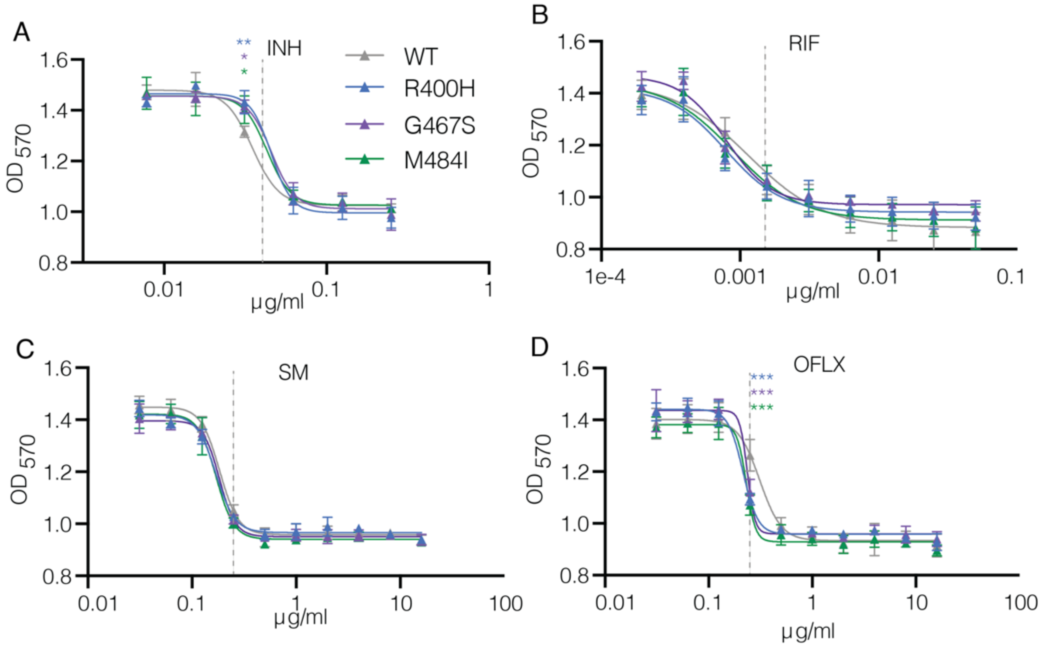

Figure S3. Drug resistance screening with alamar-blue. Alamar blue MIC measurements of indicated WT and dnaA mutant strains against INH (A), RIF (B), SM (C), and OFLX (D). Each point represents the mean and standard deviation among three independent clones for each genotype. The dashed line in each panel indicates the concentration used for competition assays in Figure 4.3. Differences among Alamar blue reduction (OD570) between WT and each mutant were tested using Dunnett's multiple comparison test after 2-way ANOVA. * $p < 0.05$, ** $p < 0.01$, *** $p < 0.001$, **** $p < 0.0001$.
