# Supplementary material for: Mutations in dnaA and a cryptic interaction site increase drug resistance in Mycobacterium tuberculosis
Source: PLoS Pathog. 2020 Nov 30;16(11):e1009063. doi: 10.1371/journal.ppat.1009063 (PMC7738170; doi:10.1371/journal.ppat.1009063)
Supplement: S4 Fig — Growth of indicated strain in the presence of varying concentrations (μg/ml) of sulfamethoxazole (A), para-amino salicylic acid (B), or pyrazinamide (C) as measured by OD600 after 6 days of growth. INH 0.04ug/ml was also tested as a control. Bars represent the mean and standard deviation of two replicates. Difference in means were tested by Dunnett’s multiple comparison test after two-way ANOVA. **** p < 0.0001 (PDF) [file ppat.1009063.s004.pdf]

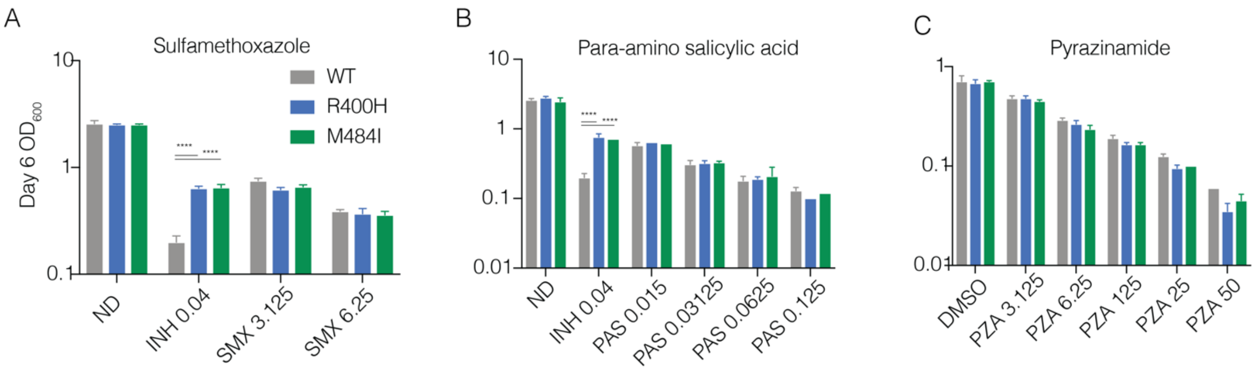

Figure S4. Drug resistance screening by OD measured growth. Growth of indicated strain in the presence of varying concentrations (μg/ml) of sulfamethoxazole (A), para-amino salicylic acid (B), or pyrazinamide (C) as measured by OD600 after 6 days of growth. INH 0.04ug/ml was also tested as a control. Bars represent the mean and standard deviation of two replicates. Difference in means were tested by Dunnett's multiple comparison test after two-way ANOVA. **** $p < 0.0001$
