# Supplementary material for: Mutations in dnaA and a cryptic interaction site increase drug resistance in Mycobacterium tuberculosis
Source: PLoS Pathog. 2020 Nov 30;16(11):e1009063. doi: 10.1371/journal.ppat.1009063 (PMC7738170; doi:10.1371/journal.ppat.1009063)
Supplement: S5 Fig — Drug concentrations indicated are in μg/ml. (PDF) [file ppat.1009063.s005.pdf]

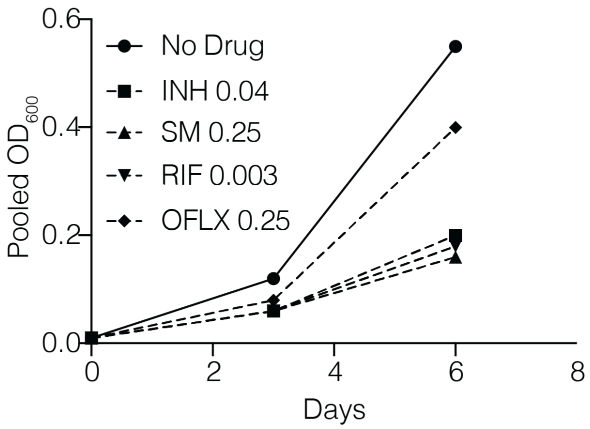

Figure S5. Bulk growth measurement of the *dnaA* barcoded library. Drug concentrations indicated are in µg/ml.
