# Supplementary material for: Mutations in dnaA and a cryptic interaction site increase drug resistance in Mycobacterium tuberculosis
Source: PLoS Pathog. 2020 Nov 30;16(11):e1009063. doi: 10.1371/journal.ppat.1009063 (PMC7738170; doi:10.1371/journal.ppat.1009063)
Supplement: S6 Fig — (A) Growth of indicated strains measured by OD600 in standard 7H9 OADC broth conditions. (B) Growth of the same strains measured by colony forming units. (C) The ratio of ori-proximal DNA to ter-proximal DNA from rapidly growing strains. (A,B) The mean and standard deviation of the three biologically independent strains measured in technical duplicate is shown. (C) Each dot represents a biologically independent strain with the mean and standard deviation shown for a genotype. Differences tested by Holm-Sidak’s multiple comparison test for indicated comparisons after one-way ANOVA. (PDF) [file ppat.1009063.s006.pdf]

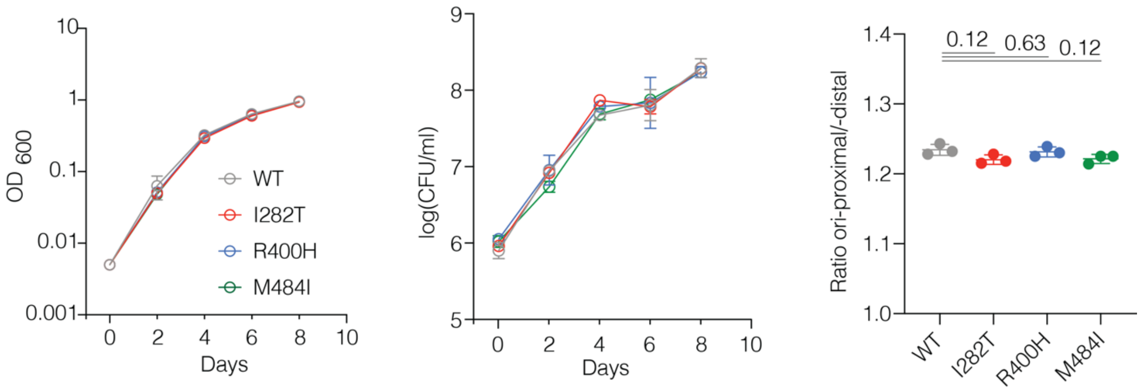

Figure S6. Growth curves and ori/ter ratio for dnaA mutants. (A) Growth of indicated strains measured by OD_{600} in standard 7H9 OADC broth conditions. (B) Growth of the same strains measured by colony forming units. (C) The ratio of ori-proximal DNA to ter-proximal DNA from rapidly growing strains. (A,B) The mean and standard deviation of the three biologically independent strains measured in technical duplicate is shown. (C) Each dot represents a biologically independent strain with the mean and standard deviation shown for a genotype. Differences tested by Holm-Sidak's multiple comparison test for indicated comparisons after one-way ANOVA.
