# Supplementary material for: Mutations in dnaA and a cryptic interaction site increase drug resistance in Mycobacterium tuberculosis
Source: PLoS Pathog. 2020 Nov 30;16(11):e1009063. doi: 10.1371/journal.ppat.1009063 (PMC7738170; doi:10.1371/journal.ppat.1009063)
Supplement: S7 Fig — INH is included as a positive control. (A) Final OD600 of the bulk culture after 6 days of growth. Each dot represents and independent culture with the mean and standard deviation shown. (B-F) Relative abundance of each strain normalized to input library. Dots represent mean normalized abundance and bars show the standard deviation of three replicates. (PDF) [file ppat.1009063.s007.pdf]

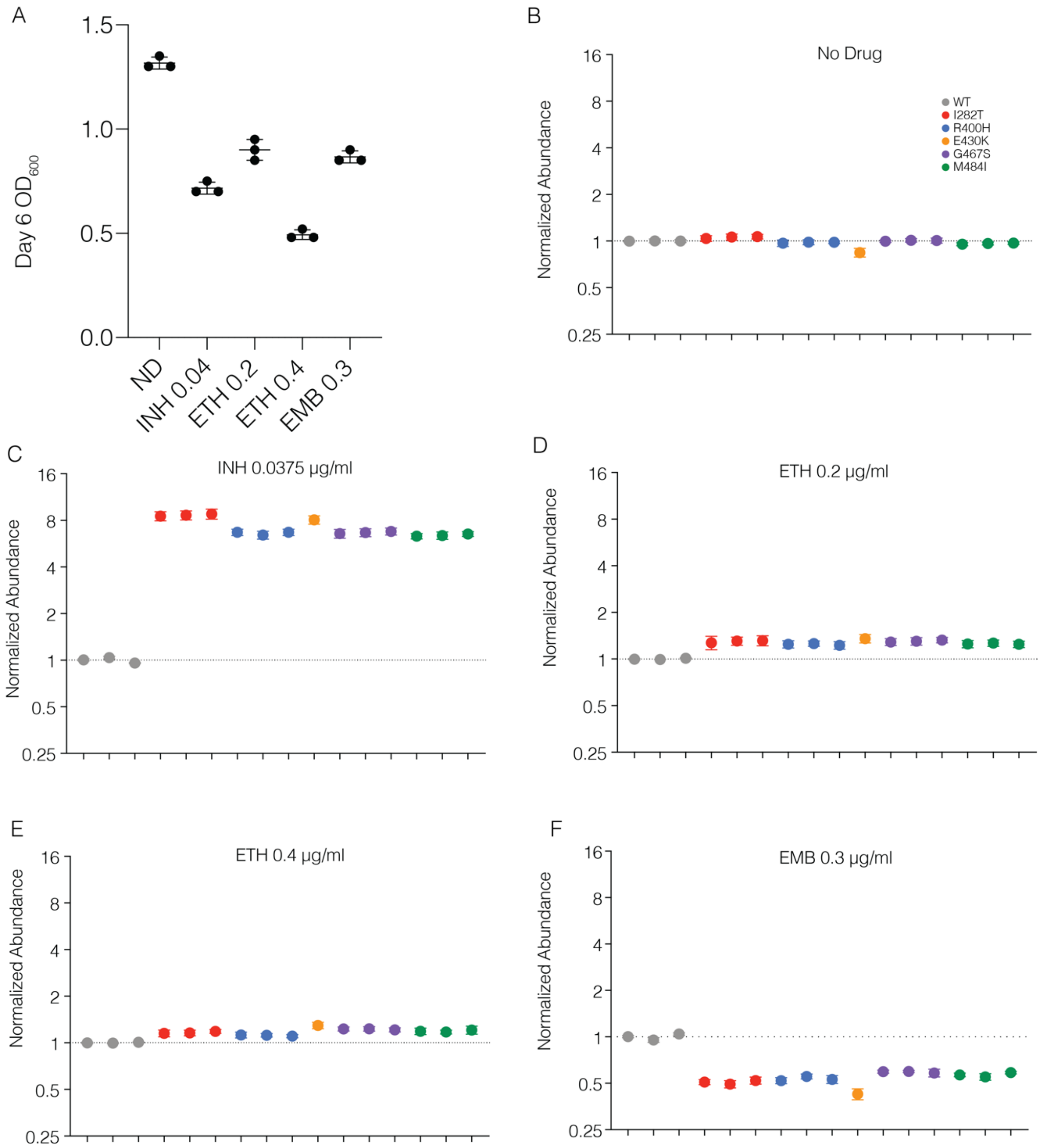

Figure S7. Competition of dnaA mutants during treatment with partially inhibitory concentrations of ethionamide (ETH) and ethambutol (EMB). INH is included as a positive control. (A) Final OD600 of the bulk culture after 6 days of growth. Each dot represents and independent culture with the mean and standard deviation shown. (B-F) Relative abundance of each strain normalized to input library. Dots represent mean normalized abundance and bars show the standard deviation of three replicates.
