# Supplementary material for: Mutations in dnaA and a cryptic interaction site increase drug resistance in Mycobacterium tuberculosis
Source: PLoS Pathog. 2020 Nov 30;16(11):e1009063. doi: 10.1371/journal.ppat.1009063 (PMC7738170; doi:10.1371/journal.ppat.1009063)
Supplement: S8 Fig — (A) Induction of dnaA protein production in E. coli BL21 cells for purification. Cells were boiled with Laemmli buffer, run on a 4–12% BIS-TRIS gel and visualized using SimplyBlue SafeStain (Thermofisher). (B) Purified dnaA protein elution fractions. The boxed protein purifications (50mM imidazole elution fraction) were used for experiments shown in Fig 4. (PDF) [file ppat.1009063.s008.pdf]

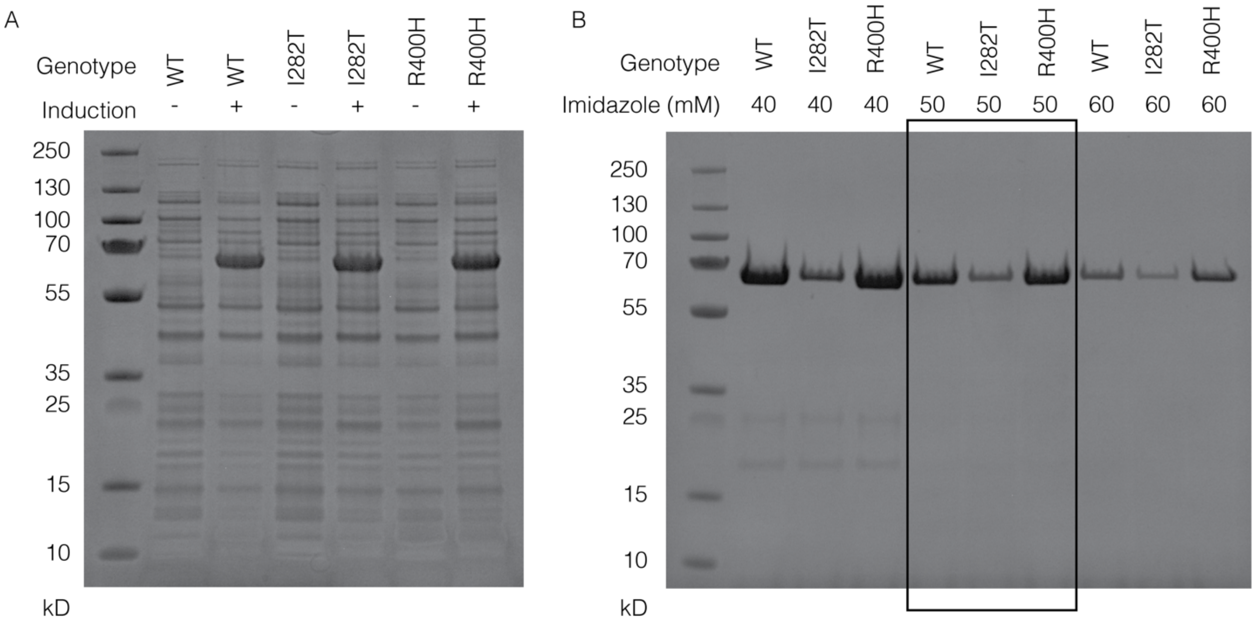

Figure S8. Purification of recombinant 6xHIS-dnaA proteins. (A) Induction of dnaA protein production in E. coli BL21 cells for purification. Cells were boiled with Laemmli buffer, run on a 4-12% BIS-TRIS gel and visualized using SimplyBlue SafeStain (Thermofisher). (B) Purified dnaA protein elution fractions. The boxed protein purifications (50mM imidazole elution fraction) were used for experiments shown in Figure 4.
