# Supplementary material for: Mutations in dnaA and a cryptic interaction site increase drug resistance in Mycobacterium tuberculosis
Source: PLoS Pathog. 2020 Nov 30;16(11):e1009063. doi: 10.1371/journal.ppat.1009063 (PMC7738170; doi:10.1371/journal.ppat.1009063)
Supplement: S9 Fig — Specific competitors for each probe were the same DNA sequence without a label. Non-specific competitor is an unlabeled fragment of sigA with the same sequence as the labeled control in Fig 4A and Fig 4C. All competitors were added at 20nM final concentration during the binding reaction.` (PDF) [file ppat.1009063.s009.pdf]

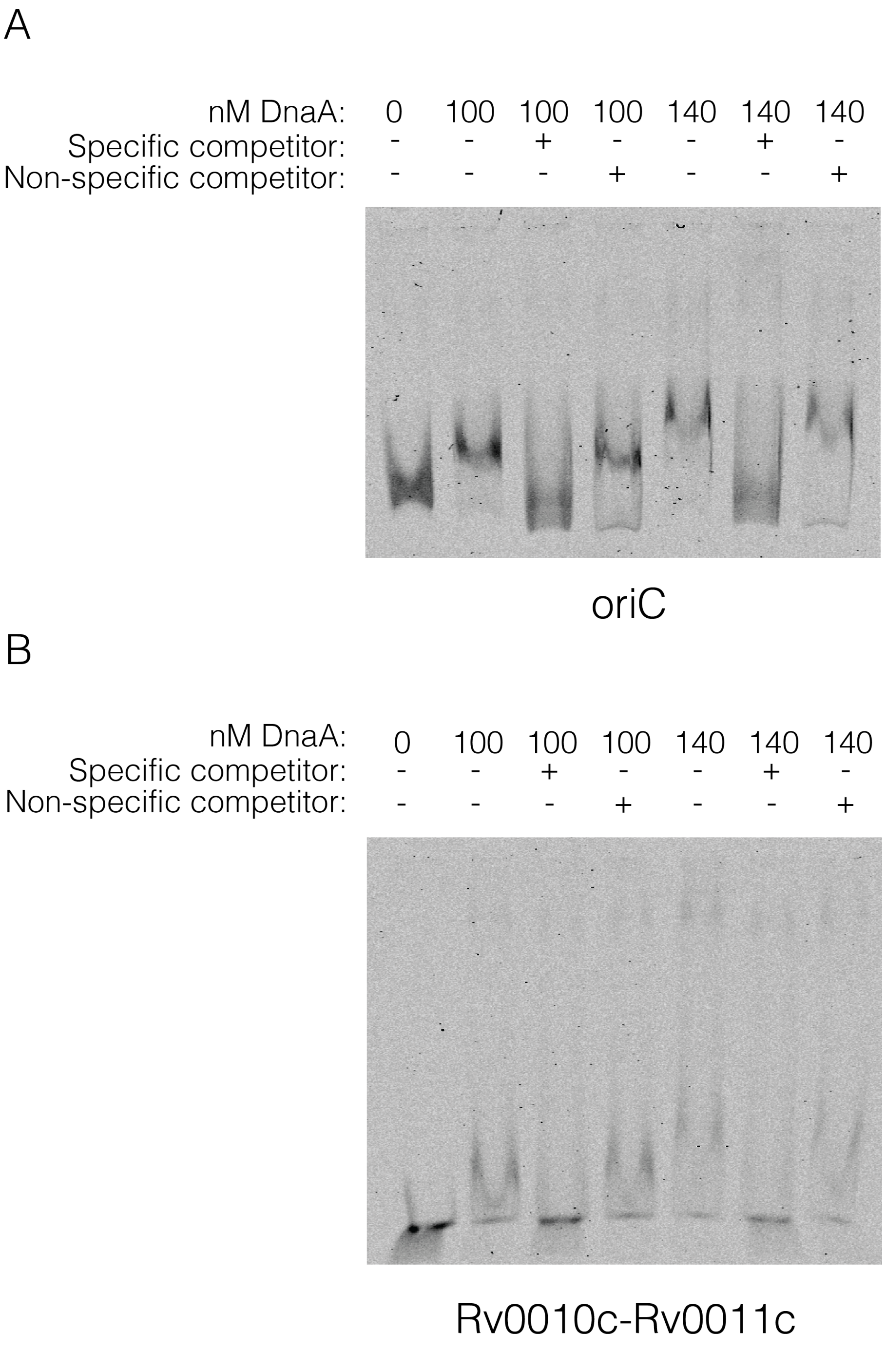

Figure S9. Competitive controls for (A) *oriC* and (B) *Rv0010c-Rv0011c* binding. Specific competitors for each probe were the same DNA sequence without a label. Non-specific competitor is an unlabeled fragment of *sigA* with the same sequence as the labeled control in Figure 4A and Figure 4C. All competitors were added at 20nM final concentration during the binding reaction.
