# Supplementary material for: Mutations in dnaA and a cryptic interaction site increase drug resistance in Mycobacterium tuberculosis
Source: PLoS Pathog. 2020 Nov 30;16(11):e1009063. doi: 10.1371/journal.ppat.1009063 (PMC7738170; doi:10.1371/journal.ppat.1009063)
Supplement: S10 Fig — (A) Enrichment plots of the IDAP-seq analysis expressed as a maximum enrichment ratio compared with a negative control containing no DnaA protein within 1000 windows across the Mtb genome (see methods). Protein concentration, total genomic DNA input, and DnaA genotype in each reaction are indicated. (B) Data from A replotted showing only the first 250kb of the genome split into 1000 windows to allow visualization of the three independent highly enriched sites near the starting coordinate. (PDF) [file ppat.1009063.s010.pdf]

A

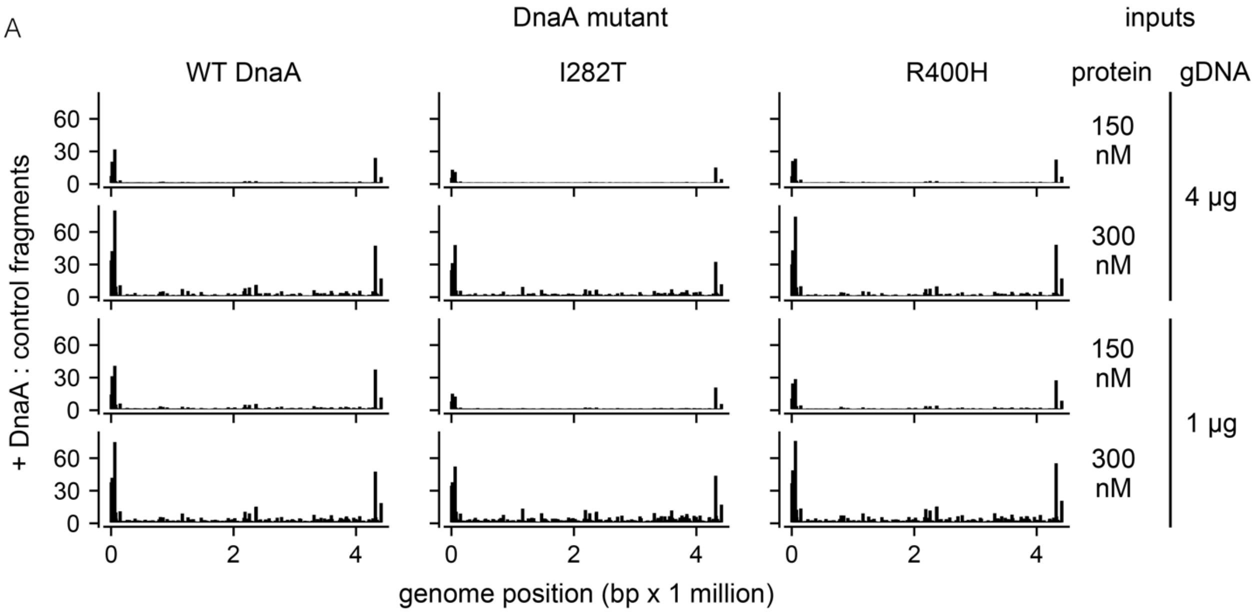


B

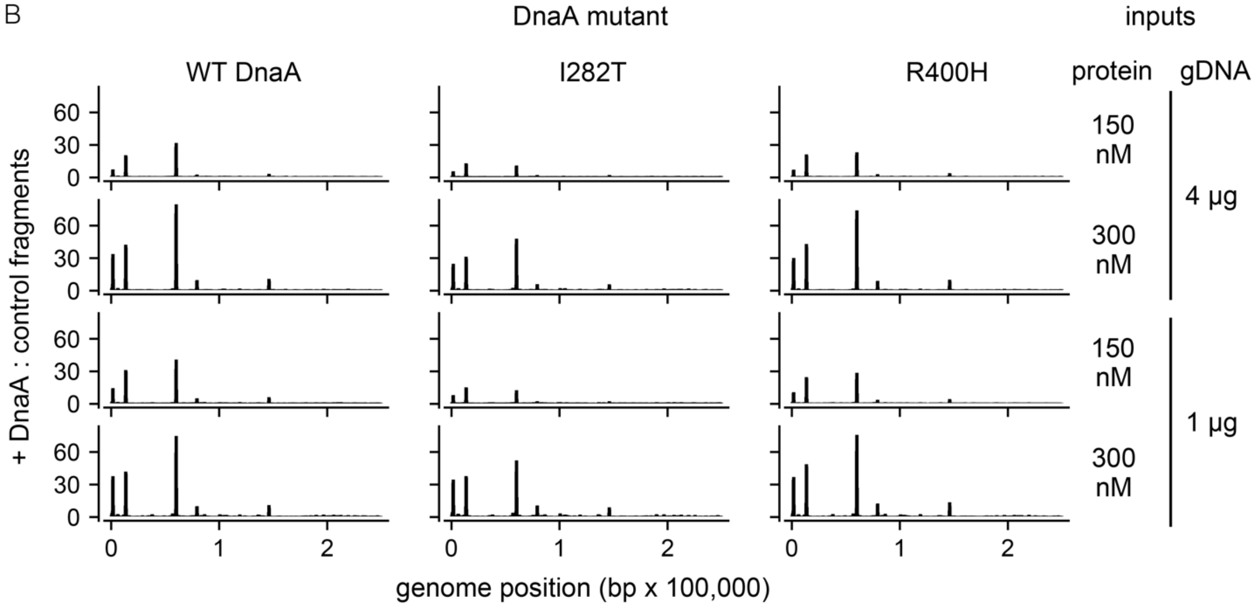


**Figure S10. Genome-wide binding of WT and mutant DnaA by IDAP-seq.** (A) Enrichment plots of the IDAP-seq analysis expressed as a maximum enrichment ratio compared with a negative control containing no DnaA protein within 1000 windows across the Mtb genome (see methods). Protein concentration, total genomic DNA input, and DnaA genotype in each reaction are indicated. (B) Data from A replotted showing only the first 250kb of the genome split into 1000 windows to allow visualization of the three independent highly enriched sites near the starting coordinate.
