# Supplementary material for: Mutations in dnaA and a cryptic interaction site increase drug resistance in Mycobacterium tuberculosis
Source: PLoS Pathog. 2020 Nov 30;16(11):e1009063. doi: 10.1371/journal.ppat.1009063 (PMC7738170; doi:10.1371/journal.ppat.1009063)
Supplement: S11 Fig — DNA sequencing coverage plots are shown for the 5 most significantly enriched regions as determined by MACS2 analysis (see methods) as visualized in Artemis (https://www.sanger.ac.uk/science/tools/artemis). The red represents sequencing depth from a myc-tagged strain and the blue line represents sequencing depth from an untagged strain used as a negative control. The fold-enrichment values from MACS2 are annotated onto each plot. Mtb homologs of M. smegmatis genes are shown below each panel. (PDF) [file ppat.1009063.s011.pdf]

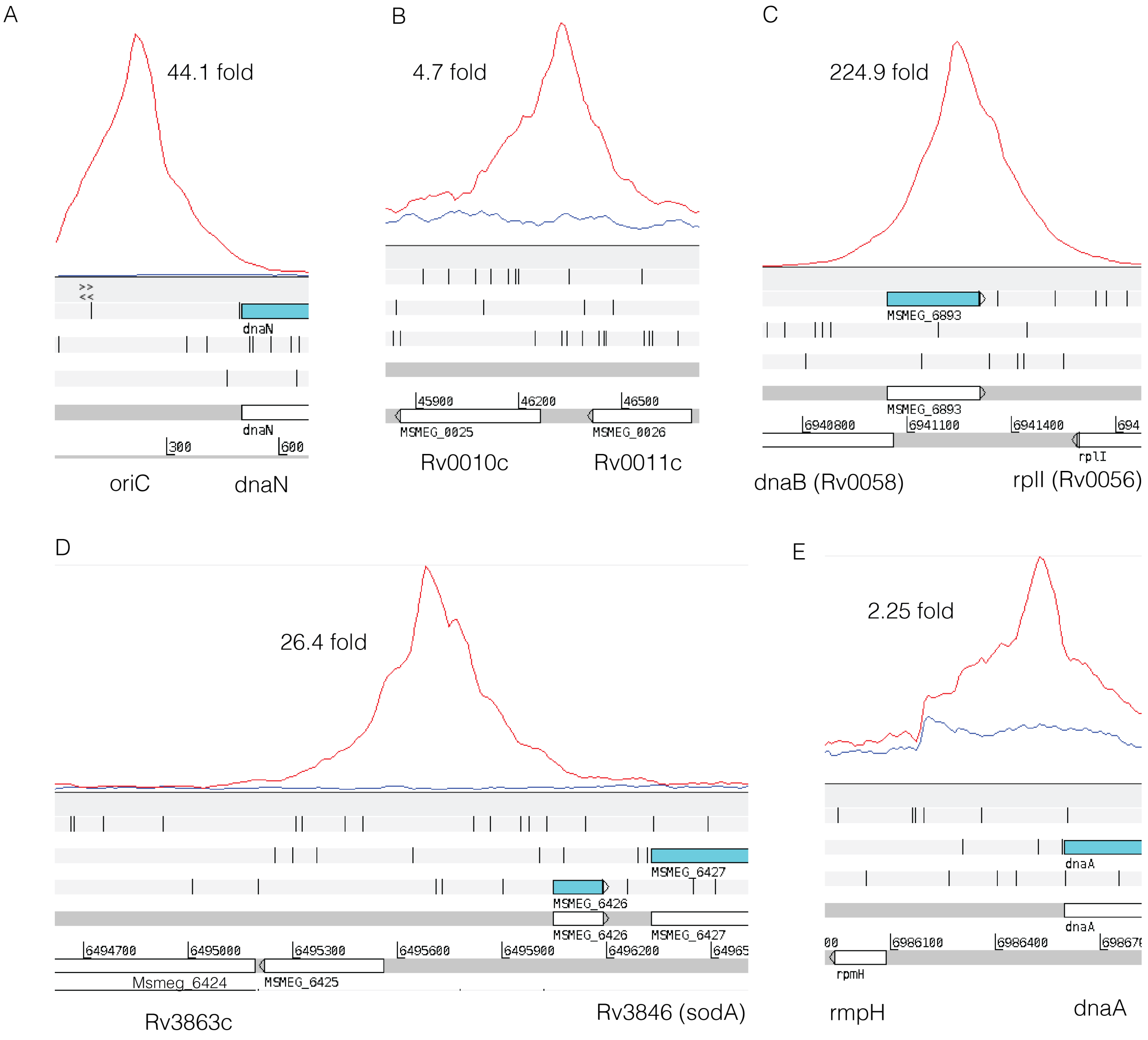

Figure S11. ChIP-seq of myc-DnaA in M. smegmatis. DNA sequencing coverage plots are shown for the 5 most significantly enriched regions as determined by MACS2 analysis (see methods) as visualized in Artemis (https://www.sanger.ac.uk/science/tools/artemis). The red represents sequencing depth from a myc-tagged strain and the blue line represents sequencing depth from an untagged strain used as a negative control. The fold-enrichment values from MACS2 are annotated onto each plot. Mtb homologs of M. smegmatis genes are shown below each panel.
