# Supplementary material for: Mutations in dnaA and a cryptic interaction site increase drug resistance in Mycobacterium tuberculosis
Source: PLoS Pathog. 2020 Nov 30;16(11):e1009063. doi: 10.1371/journal.ppat.1009063 (PMC7738170; doi:10.1371/journal.ppat.1009063)
Supplement: S12 Fig — Dashed line represents the median of the dataset. Gray lines between dots indicate each independent phylogenetic contrast. Difference in distribution tested by two-tailed Wilcoxon matched-pairs signed rank test. (PDF) [file ppat.1009063.s012.pdf]

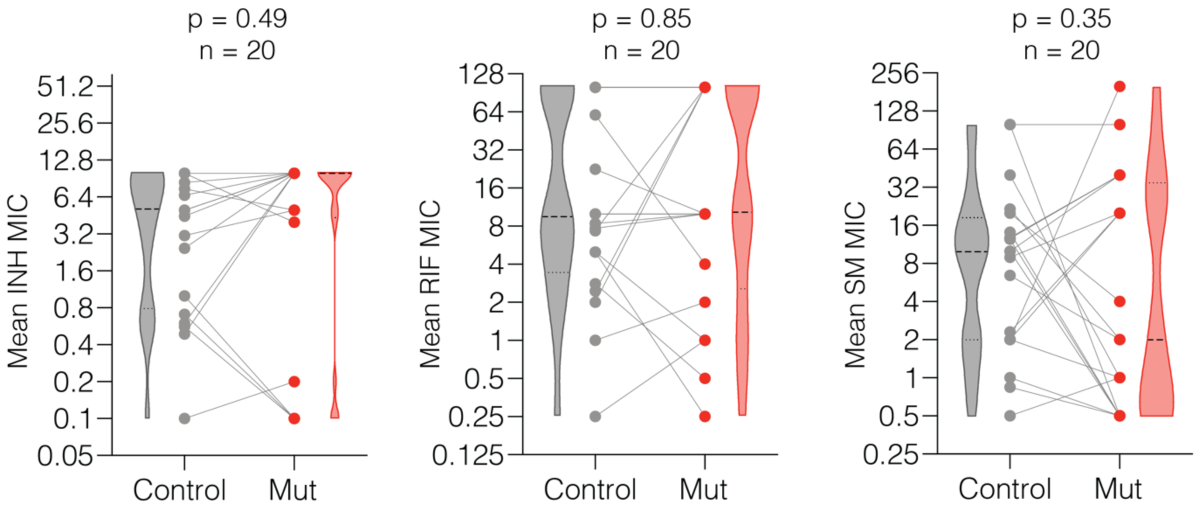

Figure S12. Comparison of the mean MIC of phylogenetic clades bearing any *Rv0010c-Rv0011c* mutation (red dots) with their nearest non-mutant neighbors (gray dots). Dashed line represents the median of the dataset. Gray lines between dots indicate each independent phylogenetic contrast. Difference in distribution tested by two-tailed Wilcoxon matched-pairs signed rank test.
