# Supplementary material for: Mutations in dnaA and a cryptic interaction site increase drug resistance in Mycobacterium tuberculosis
Source: PLoS Pathog. 2020 Nov 30;16(11):e1009063. doi: 10.1371/journal.ppat.1009063 (PMC7738170; doi:10.1371/journal.ppat.1009063)
Supplement: S1 Fig — The tree is rooted using Mycobacterium canettii as an outgroup. The inner ring depicts strains with non-synonymous dnaA mutations while each successive ring shows isolates containing a mutation shared by multiple strains. Mutations that were constructed into H37Rv are shown as stars. (PDF) [file ppat.1009063.s001.pdf]

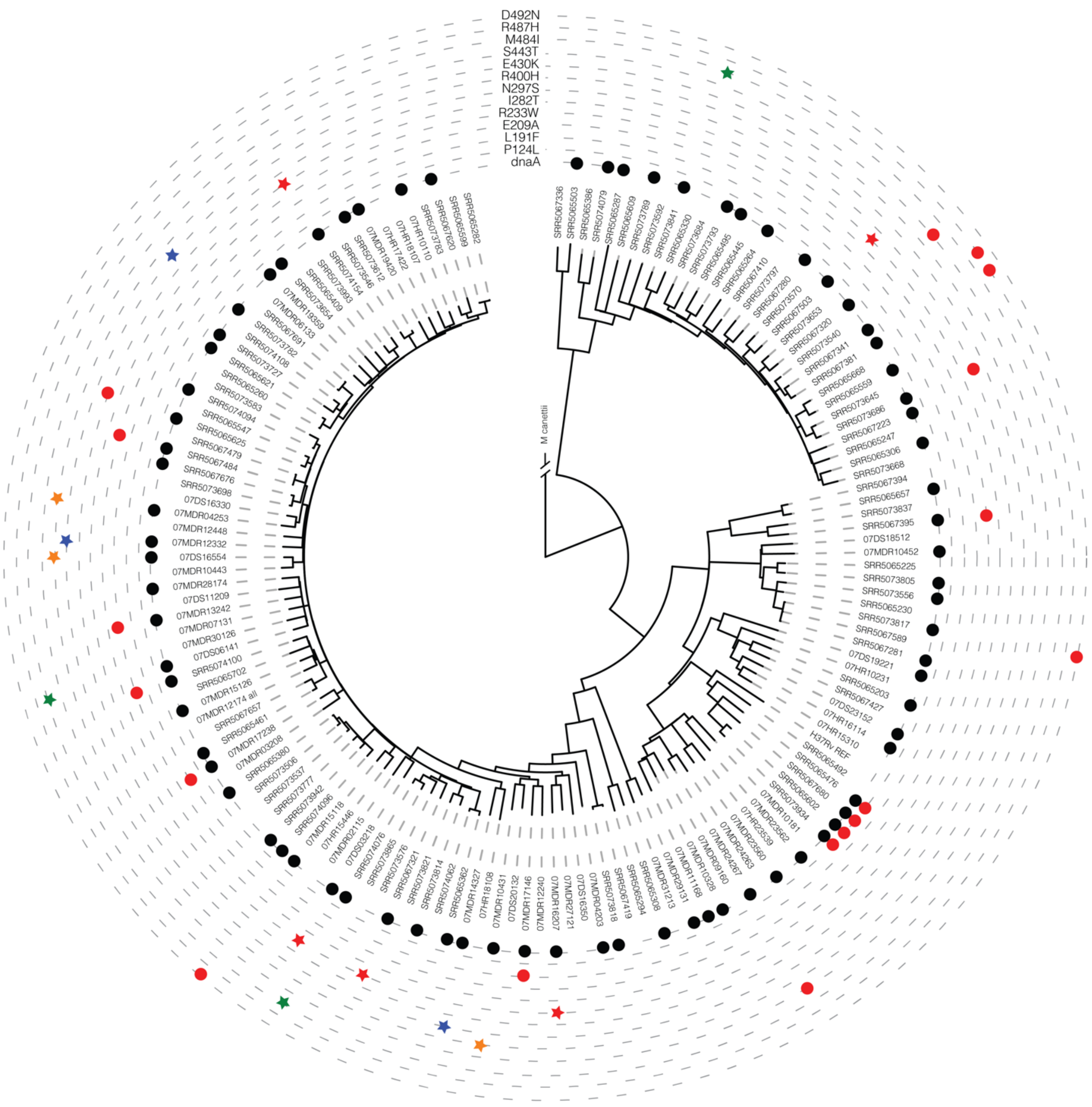

Figure S1. Whole-genome SNP phylogeny of all 78 strains containing non-synonymous dnaA mutations and their neareast, non-mutant phylogenetic neighbor. The tree is rooted using *Mycobacterium canettii* as an outgroup. The inner ring depicts strains with non-synonymous *dnaA* mutations while each successive ring shows isolates containing a mutation shared by multiple strains. Mutations that were constructed into H37Rv are shown as stars.
