# Supplementary material for: Mutations in dnaA and a cryptic interaction site increase drug resistance in Mycobacterium tuberculosis
Source: PLoS Pathog. 2020 Nov 30;16(11):e1009063. doi: 10.1371/journal.ppat.1009063 (PMC7738170; doi:10.1371/journal.ppat.1009063)
Supplement: Attachment — Submitted filename: Response to Reviewers 100620.docx [file ppat.1009063.s029.docx]

**Response to Reviewers**

*Major issues*

Reviewer 1:

1. EMSA assays are missing cold-competitive controls. These are standard in the field and should be included, especially with transcriptional regulator-type proteins

We agree. We performed the EMSA experiments again using *dnaA* at two concentrations shown to cause a shift for both the original *oriC* and *Rv0010c-Rv0011c* experiment. Addition of the non-labeled specific competitor removes the shift, while addition of the same quantity of a non-labeled non-specific competitor minimally affects binding. We have included these controls as Supplemental Figure 9 and renumbered the subsequent Supplemental Figures. We have referenced the controls in the results at the appropriate place and included the setup of this experiment in the methods section.

**Figure S9.** Competitive controls for *oriC* and *Rv0010c-Rv0011c* binding. Specific competitors for each labeled probe were the same DNA sequence without a label. Non-specific competitor is an unlabeled fragment of *sigA* with the same sequence as the labeled control in Figure 4A and Figure 4C lower panels. All competitors were added at 20nM final concentration during the binding reaction.

Reviewer 2:

1. The authors state in the abstract that “a DnaA interaction site… is under selection in clinical strains.” Homoplastic mutations are reported at this site (lines 300-310), but I do not see a formal assessment of whether more homoplasies are observed that is expected by chance. This should be done. In addition, it would in my opinion be more accurate to say the sites were subject to repeated mutation or to specifically describe the type of selection the authors speculate is at work here.

Thank you for pointing this out. We have edited the abstract to read “a DnaA interaction site that is the target of recurrent mutation in clinical strains”. To the question of whether we observe more homoplasies than expected by chance, we reexamined our strain set comprising all of the *Rv0010c-Rv0011c* mutant bearing strains and their nearest neighbor where we were able to accurately calculate homoplasies across the entire dataset of isolates from China and Vietnam. In this analysis there are 46 SNP mutation events which occur across an intergenic region of 155 bp.

To test whether some sites have more events than expected by chance, we used a simple model where the 46 mutations could occur at any site along that IG region with equal probability. We exclude INDELs here as these likely are not good assumptions (especially for *IS6110* insertions which are likely non-random). We then simulated this 100,000x and asked how often do you see ANY site with >= n independent events. For any one site among all 155 to have two independent events this is ~ p = 1.0. So not unexpected to see some homoplasy at some sites. The probability of seeing 4 or more independent events at a site is 0.035 while for sites with 6 or more this is 0.00007. This value is likely a conservative estimate of how unlikely the *Rv0010c-Rv0011c* mutation pattern is as we observe multiple sites with this level of homoplasy.

We have added text into the results to indicate this result:

Several SNP sites were significantly more homoplasic than expected by chance under a null expectation that mutations could occur with equal probability at any site in the *Rv0010c-Rv0011c* intergenic region (>=6 events p = 0.00007, see methods).

We have also explained this model and statistical test in the methods:

To statistically test whether mutations in *Rv0010c-Rv0011c* were more homoplasic than expected by chance, we compared the observed data against a null model where SNP mutation events (n=46 events) could occur at any position in the intergenic region (n=155 sites) randomly. We simulated the null distribution by randomly drawing 46 mutations from 155 sites with replacement and determined the maximum number of events that occurs at any site. We repeated this operation 100,000 times. The reported p-value is the fraction of times where six or more events occurred at any single site.

2. It’s not clear how the GWAS was done on the Vietnamese sample as the resistance phenotypes are described as being predicted in silico. In that case, the analysis would be an assessment of co-occurrence of dnaA mutations and known INH conferring mutations. Please clarify.

The reviewer is correct and we should make this more clear in the text. The GWAS with the samples from Vietnam was performed using known resistance mutations as a proxy for phenotypic resistance. The mathematical underpinning of the GWAS remains the same, but the ‘phenotype’ we associate mutations in *dnaA* or *Rv0010c-Rv0011c* with is presence of the mutation. We have amended the section describing this analysis for *dnaA* in the results to read:

To test the robustness of the association between *dnaA* variants and resistance, we examined an additional cohort of 1635 strains from Vietnam (Supplementary Table 1)*(25)* Phenotypic data was not available for this cohort so we first predicted the breakpoint resistance profiles for INH and SM *in silico.* In this independent strain collection, non-synonymous *dnaA* mutations were also statistically correlated with *in silico* predicted INH resistance (Figure 1B, *phyOverlap test*, p = 0.015) and SM resistance (p = 0.003).

And the section on Rv0010c to read:

Among the regions bound by *dnaA*, *Rv0010c-Rv0011c* is notable, as mutations in this region trended toward an association with drug resistance in clinical strains from China (INH p = 0.1, SM p = 0.06) and were highly associated with *in silico* predicted SM resistance in the cohort from Vietnam (p=0.003).

3. Line 201: The overlap among genes differentially expressed in dnaA mutants is described as “relatively high”. Please provide a formal statistical assessment of whether or not there is more overlap than is expected by chance.

Thank you for the suggestion. We agree this should be statistically tested. To address this, we performed a simulation to test the overlap among each pair of sets and the overlap of all three sets together (47 genes). In each permutation, for each strain, we randomly drew the number of genes identified as significant in our analysis (I282T n=113, R400H n=68, M484I n=63) from the entire set of features statistically tested in our RNAseq analysis (n=3976). We than calculated the degree of overlap among each pair of strains and among all three strains. We repeated this process 50,000 times. In each case, we did not in 50,000 permutations detect overlaps as high as those observed demonstrating the p-value for an overlap this high is < 1/50000 (2e-5).

We have amended the text in the results to read:

The overlaps between each pair of strains and among all three strains together were significantly higher than expected by chance (p < 2x10^-5^ in all comparisons, see methods) with 47 genes significantly dysregulated in all three mutants (Figure 3B, Supplementary Table 6)

We have also updated the methods with a description of this permutation test:

We performed a permutation test to whether the overlap among each pair of sets and the overlap of all three sets together (47 genes) was greater than expected by chance. In each permutation, for each strain, we randomly drew the number of genes identified as significant in our analysis (I282T n=113, R400H n=68, M484I n=63) from the entire set of features statistically tested in our RNAseq analysis (n=3976). We than calculated the degree of overlap among each pair of strains and among all three strains. We repeated this process 50,000 times. The p-value was the number of times we observed an overlap as high as the actual overlap in that comparison divided by 50,000.

4. Geographic patterns of dnaA mutations are shown in Figure 5 that reveal marked differences in regional prevalence. What do the authors make of these patterns? Is there any relationship between dnaA mutations and prevalence of MDR-TB? What do the authors make of the fact that dnaA mutations appear uncommon in Russia, where MDR-TB is common?

We agree that the heterogeneous distribution of the *dnaA* mutations in presumed DS-TB is surprising. We think this is likely the result of a complex set of selective factors leading to the emergence of intermediate resistance. One could imagine that strain background, multidrug regimen, patient adherence, or patient physiology could all contribute to the evolution and maintenance of these alleles in a population. We hesitate to use our dataset to comment on an overall correlation of *dnaA* prevalence and MDR-TB among countries. Many of the underlying whole-genome sequencing datasets are specifically enriched for DR-TB which impairs our ability to ask the question you raise. Russia is potentially an interesting case where although there is a high rate of MDR-TB, many of the strains in large datasets appear to be large outbreak clones (see Casali et al, Nature Genetics 2014 for an example). Thus in some cases a high MDR rate is not necessarily indicative of allowing many strains to escape and become MDR (which could be facilitated by intermediate resistance alleles) but rather by the ongoing transmission of resistant strains. We hope that a future study comparing rates of *de novo* MDR acquisition estimated from epidemiology and presumed intermediate resistance mutations is possible.

5. It’s not clear how the authors called short INDELs in dnaA (and Rv0010c-0011c).

Thank you for pointing this out. We forgot to include this in our methods. In brief, short INDELs were called using the Genome Analysis Toolkit (GATK). DnaA did not contain any INDELs in any strain in our analysis. Rv0010c-Rv0011c contained two small insertions in strains as indicated in Figure 4D. The detection of the larger *IS6110* element was performed as described in the methods section.

Reviewer 3:

1. Figures 1C and 1D present analyses of the locations of the different non-synonymous SNPs identified in dnaA. As the authors point out, the different domains (especially domains III and IV) are highly conserved: however, the sequence-level analyses in panel D appear to indicate that most of the mutations found in the MTB isolates occur in regions that are least conserved when comparing mycobacteria with E. coli and B. subtilis (as inferred from the dashed lines).

Thank you for bringing this to our attention. It is true that several of the mutations occur at local minima. To explore this, we went back to the original alignment and checked the 13 codons which are targets of convergent evolution. We have now included this as Supplemental Figure 11 where the 13 codons are boxed for readers to examine. Interestingly, although 12/13 are conserved in *M. smegmatis*, only 5 are conserved by identity with either *E. coli or B. subtilis.* 6 sites share similarity with at least *E. coli* or *B. subtilis* and 2 (including the I282T which mutates the most frequently in our data) are not conserved in either. Thus it appears that these sites are slightly more tolerant of variation than the residues around them. We have included this as Supplemental Figure 2 and updated the text to read:

Most of the *Mtb* nonsynonymous variants occur within the regulatory ATPase domain (III) or DNA binding domain (IV) (Figure 1C). These domains exhibit high sequence conservation among both bacteria generally and mycobacteria in particular (Figure 1D). However, while 12 of the 13 convergently evolved codons are conserved between *Mtb* and the environmental bacterium *M. smegmatis,* only one (G467) was absolutely conserved with *Escherichia coli* and *Bacillus subtilis* suggesting these sites may be more permissive to mutation when compared with the rest of the domain (Supplementary Figure 11).

2. In Figure 1 and throughout the manuscript: it is critical for the interpretation of the results that the authors indicate clearly whether all analyses are limited solely to non-synonymous SNPs and mutations that alter (or disrupt) the amino acid sequence; that is, all synonymous mutations are excluded. This is especially the case given that all functional validations presented in the manuscript are limited to non-synonymous mutations.

Thank you for pointing this out. We indicated that the GWAS analysis for *dnaA* relies on non-synonymous (NS) mutations, however in some figures and other places we did not make this clear. We have updated the abstract, figure 1 legend, supplementary figure 1 legend, and the discussion of the Rv0010c-Rv0011c GWAS where all mutations are included (including the INDELs) since this is an intergenic region.

3. The authors note (Lines 291-5) that dnaA and Rv0010c-Rv0011c mutations were associated with streptomycin resistance in the Chinese and Vietnamese cohorts.
   a. How is this observation reconciled with the apparent absence of a competitive growth advantage (or alteration of streptomycin MIC) in the engineered dnaA mutants?
   b. Similarly, why was the impact of Rv0010c-Rv0011c on streptomycin MIC and competitive growth advantage not determined (in Fig. 4)?
   c. Finally, given the apparent association in clinical isolates of Rv0010c-Rv0011c mutations with streptomycin resistance, what is the rationale for looking at INH susceptibility and, moreover, how is altered INH explained?

In exploring the effect of *dnaA* mutations on drug resistance, we decided to empirically test a wide panel of drugs on the assumption that GWAS against high-level resistance could be confounded by the imperfect correlation between high-level resistance and the unknown phenotype of *dnaA*. Our experimental evidence strongly supports the conclusion that *dnaA* mutations affect INH resistance specifically and we think we have demonstrated that altered *katG* expression is the most parsimonious explanation. We also believe the effect on INH is also supported by the analysis of MIC level data from clinical isolates presented in Figure 5.

Nevertheless, the stronger association with high-level streptomycin resistance mutations in strains from Vietnam is a very interesting feature of the data as you point out. We think addressing this more clearly in the discussion will improve the manuscript. For both *dnaA* and *Rv0010c-Rv0011c* using GWAS against genetic proxies of resistance, we see that in the cohort of strains from Vietnam, the stronger association is with streptomycin over INH. Notably, streptomycin is used as a part of first-line therapy in Vietnam where this is less common in other countries using standard HRZE therapy (noted in the discussion now). Our interpretation is largely that the connection between stepping-stone mutations and high-level drug resistance is complex. For example, causing an intermediate phenotype to one drug might not necessarily lead more specifically to high-level resistance of that drug over any other in a multidrug regimen in a patient. In the case of *dnaA* or *Rv0010-Rv0011c* the result from Vietnam may suggest that these mutations can lead more efficiently to high-level streptomycin resistance after causing low-level INH resistance. This is actually the case for *Staphylococcus aureus* where tolerance to one drug helps facility resistance to a different drug in a multidrug regimen (10.1126/science.aay3041). This is something that should be tested in future studies but is beyond the scope of this current manuscript. We have expanded the discussion to cite this paper and address this specific point regarding the GWAS results:

For example, here *dnaA* and *Rv0010c-Rv0011c* variants are associated with multiple breakpoint resistances in China and most strongly associated with SM resistance in Vietnam, where, unlike many TB programs, SM is used as a part of first line therapy. Nevertheless, our functional genetic studies found *dnaA* mutations cause intermediate resistance to INH alone and this was consistent with published clinical MIC data where we found *dnaA* mutations specifically coincide with quantitative increases in INH resistance—but not RIF or SM resistance. The strong association of *dnaA* and *Rv0010c-Rv0011c* with high-level streptomycin resistance could indicate that intermediate resistance to a particular antibiotic may help facilitate resistance to other antibiotics, as is the case for drug-specific tolerance mutations in *Staphylococcus aureus*[43]

Specifically in response to 3b: our decision to only perform the high-resolution phenotyping for *Rv0010c-Rv0011c* mutants in INH is that an initial screening of drug phenotypes did not reveal any indication of a shift in streptomycin MIC for Rv0010c-Rv0011c mutants. Further, the purpose of this experiment was primarily to test whether these mutations phenocopy *dnaA* mutations.

*Minor Issues*

Reviewer 1:

1. Line 184 – there is a word missing, perhaps should read, “…correlated with poor treatment…”

Thank you. We added the word ‘with’

Reviewer 2:

1. Line 17 should be “is associated with poor”

Corrected

2. Line 149 missing “of” between “panel” and “first”

Corrected

3. Line 375: remove “that”

Corrected

4. Line 490- multiple mis-spellings of Mycobacterium

We have corrected these, thank you for pointing them out.

Reviewer 3:

1. The title claims that resistance is acquired via “mutation of a DnaA pathway”. The meaning of this is unclear/inaccurate (what’s a “DnaA pathway”?) and the authors should consider a more informative alternative.

Titles are difficult and we take your point. We have adjusted the title to emphasie this is an interaction site of dnaA and place that at the forefront of the title.

**Mutations in *dnaA* and a cryptic interaction site increase drug resistance in *Mycobacterium tuberculosis***

2. L17: “is associated”

Corrected

3. L107: The authors state that “thirteen codons were targets of convergent evolution” however only 12 appear to be presented in Fig S1 and Table S2

Thank you for bringing this to our attention. We intended to address this in the figure legend. The R400H residue was not captured in this model. We have now indicated that in the figure legend.

4. L108: the T845C mutation is indicated a synonymous SNP (“T845T”) in Table S2.

We appreciate the very deep read of the supplemental material. Not all reviewers take this care. We have corrected it.

5. L120 (and elsewhere): The strains are not “Chinese” or “Vietnamese”: they are M. tuberculosis bacilli isolated from individuals in those countries. COVID-19 has provided a stark example of the dangers inherent in labelling microbes with potentially demonizing nationalities.

We totally agree and have fixed this throughout the manuscript. Thank you for bringing this up with us and helping us get it corrected.

6. The value of the modelling data presented in Fig. 1E is unclear: it seems doubtful that anyone would have expected the identified mutations to impact/alter antibiotic (INH) binding. Did the authors learn nothing from this analysis that might better inform potential explanations relating to the impact on ssDNA versus dsDNA binding?

We agree that drawing a specific conclusion on drug binding is not warranted. We also wish we could say something more about the potential effects on ssDNA binding vs dsDNA binding, however as the mutations appear across multiple surfaces and near both of these molecules, the effect on one over the other is not clear. Nevertheless we believe that including this model is useful to readers to know that they *don’t* cluster into one 3-dimensional space which would have led us to text different specific biochemical hypotheses. We have more explicitly stated the relationship with *ssDNA* and *dsDNA* as:

Rather than being clustered into a single functional region or interaction surface, the sites of convergent mutation are spread throughout surfaces on both domains with several in proximity to either *ssDNA* and *dsDNA* in the composite model.

7. In testing the first-line anti-TB agents against the engineered dnaA mutants (Fig. 2 and Fig. S2), why was ethambutol not included in the panel of antibiotics assayed?

We did in fact assay EMB resistance by competition assay in partially inhibitory concentrations. These were not initially included because they were performed at the same time as the ethionamide assays presented in Supplementary Figure 6. We don’t want to include the EMB panel in the main figure since it was performed at a different time compared with that no drug control, and have added a panel to Supplementary Figure 6 and referenced this in the text.

We further found *dnaA* mutants did not have increased resistance to the second line drug ethionamide (Supplementary Figure 6) which shares the same molecular target as INH but is activated by a *katG*-independent pathway*(36)* or to another cell wall targeting drug, ethambutol (Supplementary Figure 6)

8. Fig. 2: It would be more helpful if the antibiotic concentrations used in the competition assays were presented as a function of their MICs (e.g. 0.5X; 0.75X, etc.)

We appreciate the suggestion, but believe that specifying the exact concentration is more informative. MICs reported in the literature vary widely by exact method and interpretation. As the concentrations of drugs tested are in the partially inhibitory range, the relationship to ‘MIC’ largely depends on how we would define it.

9. L184: “correlated with poor”

Corrected

10. L303: Here and elsewhere “IS6110” (italics)

We have corrected this in the text and in Figure 4.

11. Figure 5: What is a "nearest non-mutant neighbor"; specifically, how “isogenic” are these strains – that is, are genetic differences limited primarily to dnaA SNPs or are there many others? Again, as noted in comment 2 (above), are only non-synonymous mutations included in this analysis?

The strains vary from <10 SNPs to >200 SNPs apart and have many mutations besides *dnaA* although this varies in each comparison. As such we cannot definitively say that the nonsynonymous *dnaA* variants cause a specific change in any individual comparison but only that on average, *dnaA* nonsynonymous mutants have an increased MIC. We have clarified this in the text. We also make it clear that only non-synonymous SNPs are considered here.

Although multiple mutations throughout the genome distinguish each *dnaA* mutant clade and control clade, mutants had on average significantly higher resistance to INH when compared to control clades (Figure 5, median increase = 2-fold, p = 0.0038, Wilcoxon matched-pairs signed rank test two-tailed).

12. L357: It is not clear what is meant by “in silico breakpoint resistance” (L357); the authors should provide more detail about how this was determined.

We have updated the results section at L357 to specifically indicate this means the absence of known resistance conferring mutation. Those mutations referenced in the methods:

11,718 had a geographic label and were drug susceptible by *in silico* breakpoint resistance DST as determined by the absence of known resistance conferring mutations.

13. L406-8: This statement is no longer accurate, and the data presented in this manuscript should be discussed in the context of recent work from Sassetti and colleagues (doi: 10.1016/j.cub.2020.07.070).

This statement was worded too broadly thank you for pointing it out. We cited the biorxiv version of this paper on line 401 and will update that reference. Our statement is meant to indicate that we don’t really know what molecularly causes these oscillations despite seeing coherence across genes involved in particular metabolic pathways. We have updated this statement by citing Bandekar et al again and it now reads as:

However, while recent work has implicated variation in gene expression across the cell cycle with requirement of gene function, suggesting the catalase/peroxidase activity of KatG may be differently required across the cell cycle (Bandekar *et al.*), further work will be necessary to understand the link between cell cycle perturbation and altered *katG* expression.

14. Supplementary Figure 2: no labels are provided indicating which plot relates to which mutant.

Thank you for pointing this omission. We have added the legend.

*Additional editorial changes for PLOS pathogens:*

1. We have added an author summary at the front of the article.
2. We have removed the statement from the methods in which we mention our initial controls to ensure that dnaA protein binding activity is not affected by freezing. We presented this without supporting data, however, this control is not important for the interpretation of the paper.

~~Comparison of fresh and frozen protein by EMSA analysis showed no loss of DNA binding activity (data not shown).~~

3. We have added a note to Figure 5 legend the methods to state that we made the figure using the Tableau software package.
4. The final copy for submission has altered all Supplementary Table and Figure names Figure S# or Table S#.
